# Identification and Phylogenetic Analysis of the *R2R3-MYB* Subfamily in *Brassica napus*

**DOI:** 10.3390/plants12040886

**Published:** 2023-02-16

**Authors:** Dingfan Luo, Desheng Mei, Wenliang Wei, Jia Liu

**Affiliations:** 1 College of Agriculture, Yangtze University, Jingzhou 434023, China; 2 Oil Crops Research Institute, Chinese Academy of Agricultural Sciences, No. 2 Xudong 2nd Rd., Wuhan 430062, China; 3 Key Laboratory of Biology and Genetic Improvement of Oil Crops, Ministry of Agriculture and Rural Affairs, Wuhan 430062, China

**Keywords:** *Brassica napus*, drought stress, *R2R3-MYB* genes

## Abstract

The R2R3-MYB sub-family proteins are composed of most members of MYB (v-Myb avian myeloblastosis viral oncogene homolog) protein, a plant-specific transcription factor (TF) that is classified into four classes depending on the number of MYB repeats. R2R3-MYB TFs are involved in physiological and biochemical processes. However, the functions of the *Brassica napus R2R3-MYB* genes are still mainly unknown. In this study, 35 *Brassica napus MYB* (*BnaMYB*) genes were screened in the genome of *Brassica napus*, and details about their physical and chemical characteristics, evolutionary relationships, chromosome locations, gene structures, three-dimensional protein structures, cis-acting promoter elements, and gene duplications were uncovered. The *BnaMYB* genes have undergone segmental duplications and positive selection pressure, according to evolutionary studies. The same subfamilies have similar intron–exon patterns and motifs, according to the genes’ structure and conserved motifs. Additionally, through cis-element analysis, many drought-responsive and other stress-responsive cis-elements have been found in the promoter regions of the *BnaMYB* genes. The expression of the *BnaMYB* gene displays a variety of tissue-specific patterns. Ten lignin-related genes were chosen for drought treatment. Our research screened four genes that showed significant upregulation under drought stress, and thus may be important drought-responsive genes. The findings lay a new foundation for understanding the complex mechanisms of *BnaMYB* in multiple developmental stages and pathways related to drought stress in rapeseed.

## 1. Introduction

In land plants, drought is often a major stress that can reduce crop productivity severely [1]. Rapeseed plants suffer terrible consequences when there is a water shortage, resulting in changed physiological processes (photosynthesis rates, osmotic protection, and oil contents) and reduced plant growth [2]. Rapeseed is quite vulnerable to drought stress during the flowering stage, and it has been projected that global climatic oscillations, which are the cause of severe and prolonged drought in some areas of the biosphere, will reduce rapeseed output [3]. Therefore, finding the methods that can mitigate drought stress is critical for *B. napus (Brassica napus* L.). A method of accumulating secondary metabolites are formed through accumulating lignin [4,5,6,7,8,9]. In poplar (*Populus*), overexpression of the transcription factor PdNF-YB21 promotes root growth, along with drought tolerance [9]. Furthermore, overexpression of *VlbZIP30* in grapevine (*Vitis vinifera*) [8], *poCCoAOMT* in tobacco (*Nicotiana benthamiana*) [10], *IbLEA14* in sweet potato *(Ipomoea batatas)* [11], *osTF1L* in rice [12], and *PeLAC10, CmCAD2* and *CmCAD2* in Arabidopsis (*Arabidopsis thaliana*) has been found [7,12,13]. All these genes enhance the synthesis of lignin and drought resistance. Inbred lines of maize (*Zea mays*) that are more lignified than drought-sensitive lines imply that lignification is a key mechanism for drought stress adaptation [14].

There are several genes within the *MYB* gene family that act as TFs for plants. There is a conserved DBD (DNA-binding domain) in the MYB transcription factor, which can be composed of up to four imperfect repeats, known as R repeats [15,16]. Each R repeat’s second and third helices join with specific gene-specific promoter sequences to create a helix-turn-helix (HTH) structure, which is 50–55 amino acids long [15,17]. In addition, the third α-helix is usually responsible for recognizing short DNA sequences [18]. Sequence similarity determines whether the MYB domain repeats belong to the R1, R2, or R3 category [16,19]. Members of the plant MYB superfamily generally consist of one to four imperfect MYB repeats, and there are four types of repetitions: 1R-MYB (one or two separated repeats), 2R-MYB (R2R3-MYB, two adjacent repeats), 3R-MYB (three adjacent repeats), and 4R-MYB (consisting of four adjacent repeats) [16]. The R2R3-MYB family has considerably grown in size and now dominates in the plant lineage [20].

In a recent study [21], 2120 *R2R3-MYB* genes were identified in six brassica plants, including 130 in *Arabidopsis*, 236 in *B. rapa*, 247 in *B. oleracea*, 248 in *B. nigra*, 425 in *B. napus*, 422 in *B. juncea* and 412 in *B. carinata*. However, the number of *R2R3-MYBs* in *Arabidopsis*, *B. rapa* and *B. napus* were different from those found in previous studies, namely, 126 in *Arabidopsis* [18], 256 in *B. rapa* [22], and 249 in *B. napus* [23]. The main reason may be that with the development of third-generation sequencing technology, the information has become more complete.

R2R3-MYB TFs have been shown to synthesize hormones, participate in signal transduction, and be involved in physiological and biochemical processes, particularly in reaction to a variety of biotic and abiotic stimuli [16,24,25,26]. In addition, R2R3-MYB also contributes significantly to lignin synthesis [16]. The R2R3-MYB transcription factor PbrMYB169 protein of pear (*Pyrus bretschneideri*) significantly activated the promoters of lignin genes *C3H1, CCR1, CCOMT2, CAD, 4CL1, 4CL2, HCT2* and *LAC18* by binding with the AC element in the promoter, thus positively regulating the lignification in the cell matrix of pear fruit [27]. The constitutive expression of three members of MYB transcription factor family (PtrMYB92, PtrMYB3 and PtrMYB20) in poplar activated the expression of lignin biosynthesis gene and induced the ectopic deposition of lignin. At the same time, they can also activate the promoter of poplar wood biosynthesis gene [28,29]. The overexpression of TaMYB4 leads to the reduction of the transcription of *CAD* and *CCR* genes involved in the lignin biosynthesis pathway, which has a negative regulatory effect on the lignin biosynthesis in wheat (*Triticum aestivum* L.) [30]. ZmMYB31 and ZmMYB42 can downregulate the *COMT* genes of maize (*Zea mays* L.) and Arabidopsis, and overexpression results in a reduction of lignin content in transgenic plants [31]. The overexpression of MusaMYB31 can downregulate many common phenylpropane genes and lignin biosynthesis pathways, and lignin biosynthesis is negatively regulated in banana (*Musa acuminata*) [32]. According to these results, we found that the R2R3-MYB transcription factor can bind to the promoter and significantly activate the lignin gene. However, the expression of some R2R3-MYB transcription factors can downregulate the genes in the lignin synthesis pathway, thus negatively regulating the lignin biosynthesis in plants. We have learned that lignin deposition has a great impact on drought responses, so *R2R3-MYB* is responsive to drought stress while regulating lignin synthesis.

In *Arabidopsis*, there are about 126 members of the *R2R3-MYB* subfamily, which is roughly divided into 25 subgroups and distributed across 11 branches [18]. The functions of most members have been identified, and they have been found to be involved in the control of plant-specific processes, including (i) primary and secondary metabolism, (ii) cell fate and identity, (iii) development processes, and (iv) the response to biological and abiotic stresses. We found in the literature that *AtMYB26* can control the deposition of secondary walls in the anthers [33]. *AtMYB46* is a positive regulator of lignin biosynthesis in fibers and vessels [34]. These two genes may be able to cope with drought stress by regulating the production of lignin. These two genes are not included in the 25 subgroups but are in the same branch as Subgroups 13 and 16. Therefore, we preliminarily selected the Brassica napus homologs to this branch for further analysis.

In this work, 35 *BnMYB* genes of rapeseed were discovered by comparing the genomic sequences of the S13 and S16 subfamilies of the known *R2R3-MYB* from *Arabidopsis thaliana*. Additionally, we used the method of bioinformatic analysis to effectively process, analyze, and visualize a large amount of data from the field of life science. The physical and chemical characteristics of the *BnMYB* genes’ cis-acting promoter and their gene duplication were clarified, as well as their evolutionary relationships, genomic positions, gene structures, and three-dimensional membrane proteins. We also looked at how the *BnaMYB* gene family was expressed in various tissues and how they responded to drought treatment. Further research into the functional characterization of *BnMYBs* may benefit from our findings.

## 2. Results

### 2.1. Identification of the R2R3-MYB Transcription Factor Family in Rapeseed

To identify *R2R3-MYB* family genes in rapeseed, 12 AtTCP protein sequences were used as queries for a BlastP search against the rapeseed genome. As a result, 35 *BnMYB* genes were consistent with the R2R3-MYB domain (Table 1). Hereafter, these genes are named *BnMYB1* to *BnMYB35*. The physical and chemical characteristics of each member were then examined and predicted. The proteins that the *BnaMYB* genes encoded ranged in size from 239 amino acids (*BnaMYB35*) to 370 amino acids (*BnaMYB17, BnaMYB18*); the MW ranged from 77,433.37 kDa (*BnaMYB8*) to 195,053.44 kDa (*BnaMYB23*); and the PI ranged from 4.89 (*BnaMYB23*) to 5.11 (*BnaMYB8*). The predicted subcellular location of each BnaMYB protein was in the nucleus. Table 1 also contains the length of the coding sequences.

### 2.2. Location of the Chromosomes and Phylogenetic Analysis

**Figure 1:**
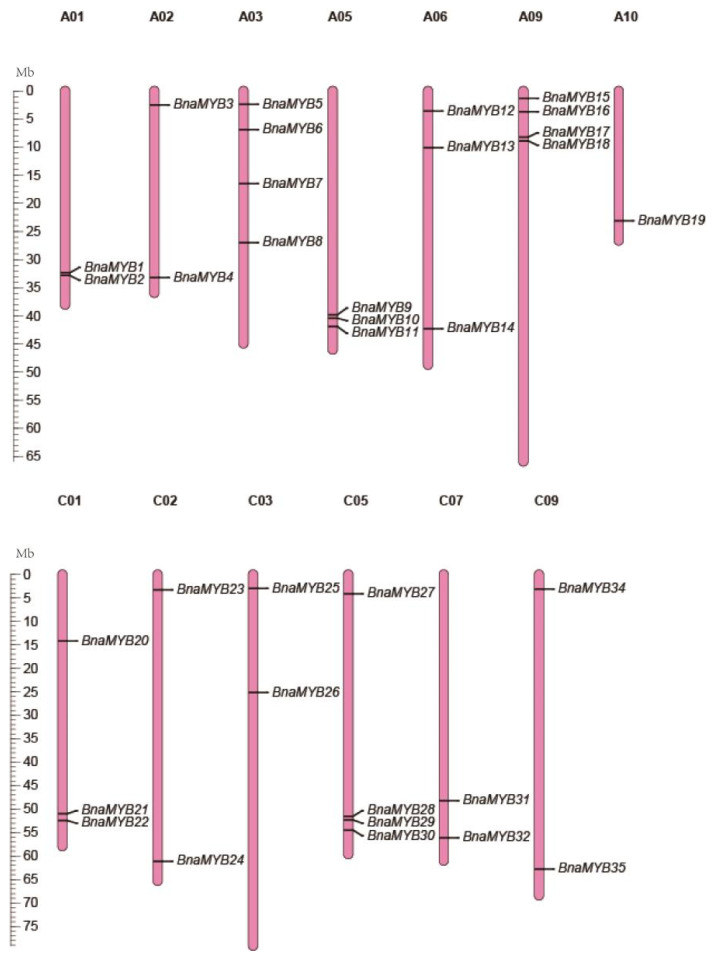
Chromosomal localization of *BnaMYB* genes in *B. napus*. The reference genome used was ZS11. The chromosomes with different sizes are represented by the pink vertical bars with different lengths. The locations of genes are shown from top to bottom.

Additionally, the 35 *BnaMYB* genes were found on the chromosomes (Figure 1). The rapeseed genome had 35 *R2R3-MYB* genes that were widely distributed throughout 19 chromosomes. On chromosome A10, there was just one gene; on chromosomes A01, A02, C02, C03, C07 and C09, there were two genes; on chromosomes A05, A06, and C01, there were three; and on chromosomes A03, A09 and C05, there were four. Table S1 shows the position of each gene on the chromosomes.

**Figure 2:**
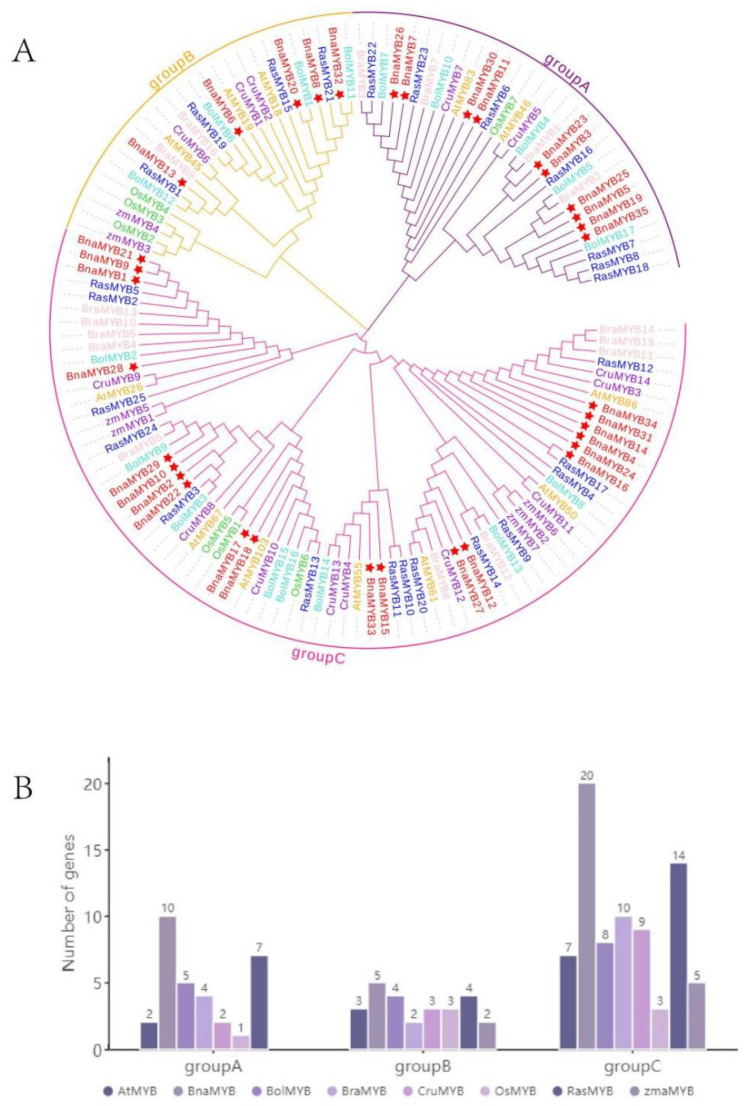
Phylogenetic analysis and number of *R2R3*-*MYB* homologs in different species. (**A**) Phylogenetic tree using 133 R2R3-MYB proteins from 7 species, including rice, maize, *Arabidopsis*, *B. napus*, *B. oleracea*, *B. rapa* and radish. The clades of Groups A, B and C are marked in purple, yellow, and pink, respectively. Among them, *BnaMYBs* are represented by red five-pointed stars. The abbreviations represent the species as follows: Os, *Oryza sativa*; Zma, *Zea mays*; At, *Arabidopsis thaliana*; Bna, *Brassica napus*; Bol, *Brassica oleracea*; Bra, *Brassica rapa*; Cru, *Capsella rubella*; Rsa, *Raphanus sativus*. (**B**) The number of *R2R3-MYB* genes in each group. The ordinate is the number of genes, and the abscissa shows the *R2R3-MYB* genes of each species.

We built a phylogenetic tree using 133 MYB proteins from seven different species, including *Arabidopsis*, *Brassica napus*, *Brassica oleracea*, *Brassica rapa*, *Capsella rubella*, *Oryza sativa*, *Raphanus sativus* and *Zea mays*, to better understand the evolutionary relationships among the *MYB* genes of the different species. Table S2 shows the protein sequences. These *MYB* genes were divided into three groups, as seen in Figure 2A. The majority of *MYB* members were found in the third group, which consisted of seven species, while the second group had the fewest *MYB* genes. Groups A, B, and C contained 10, 5 and 20 *BnaMYB* gene members, respectively (marked with asterisks in Figure 2A). Members of *BnaMYB* that clustered together may have a special connection and carry out similar functions.

The number of *R2R3-MYB* genes in each subspecies within each group was then tallied (Figure 2B). Although the first group had no examples of *ZmMYB*, the seven species were distributed throughout the three groups. Each group of species had two to four copies of the *R2R3-MYB* genes, depending on the quantity of these genes in *A. thaliana*. We found that the *BnaMYB* genes in Group A were all homologs of *AtMYB46*, and the *BnaMYB* genes in Group C were all homologs of *AtMYB86.*

### 2.3. Conserved Motifs and Gene Structure

**Figure 3:**
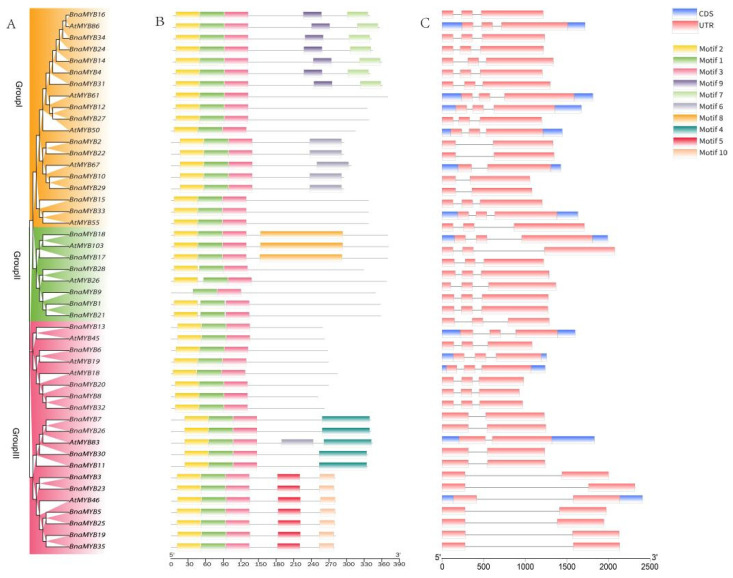
Comparative analysis of conserved domains and gene structure between *AtMYB* genes and *BnaMYB* genes. (**A**) The phylogenetic analysis of 12 *AtMYB* genes and 35 *BnaMYB* genes divided them into three groups: I, II and III. (**B**) The conserved domains of AtMYB and BnaMYB proteins. Ten conserved motifs of the BnaMYB protein were identified through the MEME website (https://meme-suite.org/meme/, accessed on 30 March 2022). Different colored boxes represent different motifs. (**C**) The gene structure of *AtMYB* and *BnaMYB* genes. The green box represents exons, the yellow box represents the UTR, and the black line represents introns. All results were visualized with TBtools software (v1.098769).

Because Arabidopsis is a model plant, the research on its genome and gene function is the most complete. Rape and Arabidopsis belong to the same family of Cruciferae, with the closest genetic relationships and the clearest comparisons of genome homology. Therefore, we created an evolutionary tree with 35 BnaMYB protein sequences from *B. napus* and 12 AtMYB protein sequences from *A. thaliana* to examine the structural variety of *BnaMYB* genes. All of the *R2R3-MYB* genes were divided into Groups I, II and III (Figure 3A). Their conserved protein domains and gene structure were then investigated further.

The full-length protein sequences were examined to identify their conserved motifs (Figure 3B). The *R2R3-MYB* genes’ conserved motifs ranged from two to five. Ten conserved motifs in all were found; their widths ranged from 26 to 141 amino acids (Table S3). In addition, individuals of the same group had common conserved motifs. For instance, Motifs 7 and 9 were particular to Class I, Motif 8 to Class II and Motifs 4, 5 and 10 to Class III. The quantity and organization of the *MYB* motifs in the various groups, however, varied slightly. In Group II, *BnaMYB9* had fewer conserved motifs than other members, whilst *BnaMYB17, BnaMYB18* and *AtMYB103* all had conserved motifs that were different from the other members.

To further understand the development of the *R2R3-MYB* gene family in rape, according to the findings, there were between one and either two or three exons and introns, respectively (Figure 3C). Sixteen genes had two exons and one intron, whereas 31 genes had three exons and two introns. Exon gains and losses were discovered during the evolution of the *MYB* family of genes. Our findings suggested that over the evolution of the rapeseed genome, the exon–intron architecture of *MYB* genes remained relatively stable. Additionally, the *BnaMYB* gene members within a class had gene architectures that were quite similar and consistent with their evolutionary groups. In summary, according to our analysis of the conserved motif composition, gene structures, and phylogenetic interactions, the consistency of the class organization was convincingly sustained, showing that the MYB proteins have extraordinarily well-maintained amino acid residues and that the members within a class may play parallel roles.

### 2.4. Multi-Sequence Alignment and Predicted 3d Protein Structures

We used 3D structure prediction analysis and multiple sequence alignment to clarify the structural properties of the BnaMYB proteins (Figures S1 and S2). These findings led us to the conclusion that the MYB DNA-binding domains in these proteins are highly conserved. The BnaMYB proteins were split into three groups on the basis of their genetic compatibility.

### 2.5. Collinearity within B. napus and among Different Species

**Figure 4:**
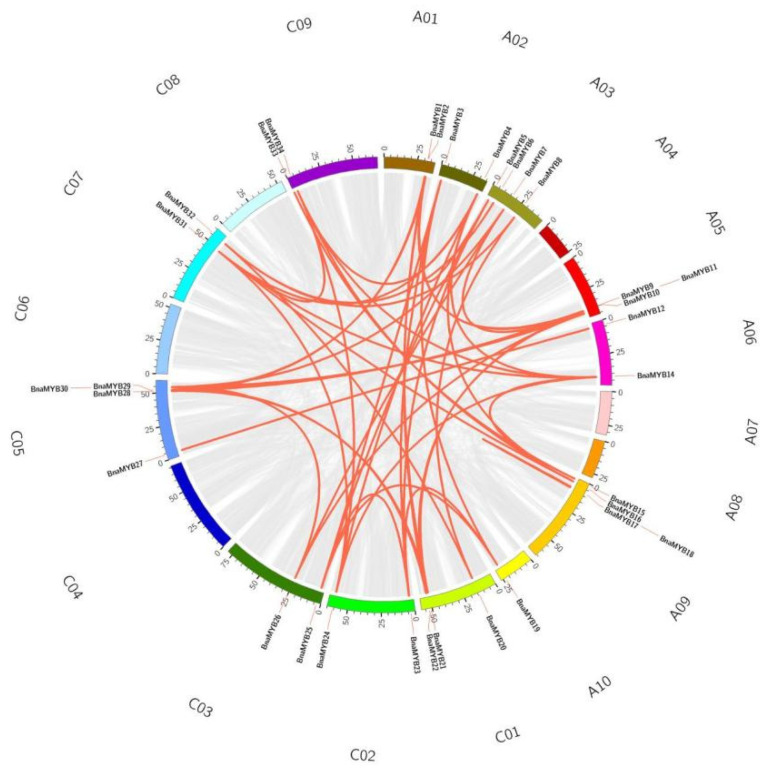
Collinearity analysis of the AC subgenome of *R2R3-MYB* genes in *B. napus*. The gray lines represent the replication events of all genes in *B. napus* and the red lines represent tandem repeat events within the *BnaMYB* genes.

Analysis of genome-wide replication is crucial for understanding the genesis, evolution, and genome-wide expansion of organisms. To further understand the reasons behind gene replication events in *BnaMYB*, we therefore examined the replication events of the *R2R3-MYB* gene family in *B. napus*. A04, A07, A08, C04, C06 and C08 were the only chromosomes without fragment repeats, along with 48 pairs of genes with large fragment repeats (Figure 4). These findings suggested that the amplification and evolution of the *R2R3-MYB* genes in the *B. napus* genome may have been significantly influenced by the replication of large fragments.

**Figure 5:**
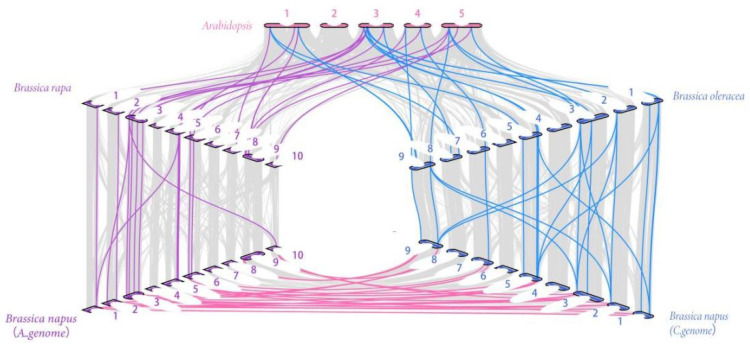
Syntenic relationships of R2R3-MYB genes in *B. napus* and three ancestral plant species. The figure shows the collinearity between *Arabidopsis* (*A. thaliana*), *Brassica rapa* (*B. rapa*), *Brassica oleracea* (*B. oleracea*) and *Brassica napus* (*B. napus*).

The evolution of the *R2R3-MYB* gene family in *Brassica* has been tracked in previous research [21]. In order to specifically understand the evolutionary relationship of target genes, we analyzed the homologous relationship among *Arabidopsis*, *B. napus* (A and C subgenomes), *B. rapa* (A genome), and *B. oleracea* (C genome) (Figure 5). The collinearity analysis of *MYB* showed that there were a large number of orthologous *MYB* genes in *Arabidopsis, B. rapa, B. oleracea*, and *B. napus.* There were 16 pairs of genes in *Arabidopsis* and *B. rapa* that showed collinearity, and 15 *B. rapa R2R3-MYB* genes had homologous genes in *Arabidopsis*, 10 of which were multi-copy genes and 5 were single-copy genes. *B. rapa* lacked *AtMYB50* homologs, indicating that gene loss occurred in *B. rapa* during evolution. Furthermore, 16 *B. oleracea R2R3-MYB* genes contained similar genes to *Arabidopsis*. The A and C subgenomes of *B. napus* mostly overlapped those of the related diploids *B. rapa* and *B. oleracea*. Fourteen similar gene pairs were discovered in the A genome of *B. napus* and *B. rapa*, and 21 in the C genome of *B. napus* and *B. oleracea*. Even if gene loss has occurred, the great majority of *R2R3-MYB* genes in *B. napus* are still present and have contributed to the evolution of the *MYB* gene family.

### 2.6. Synteny of BnMYB Genes

We compared the CDS sequences (Figure S3), which showed the similarity and identity of these sequences more clearly. We estimated the synonymous (Ks) and nonsynonymous (Ka) replacement rates (Ka/Ks) of 44 segmented repeated pairs (Table S4). The average Ka/Ks ratio of fragmented repeated gene pairs was 0.61. The calculation results showed that the Ka/Ks ratio of all duplicate BnMYB gene pairs was less than 1, which means that they are undergoing a purification selection process.

### 2.7. Cis-Acting Elements and Functional Annotation

**Figure 6:**
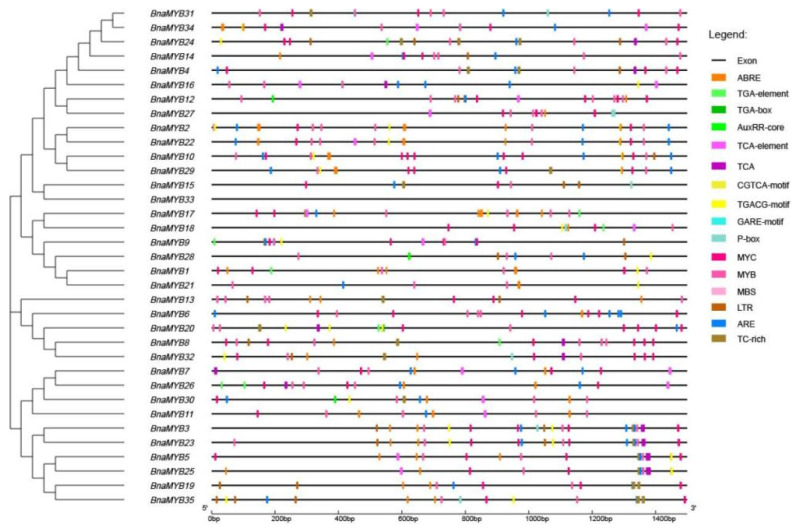
Cis-acting element analysis of *MYB* gene subfamily in *B. napus.* The boxes with different colors on the black line represent different cis-elements.

Transcriptional regulators bind to the cis-acting elements, which control gene transcription. We examined the cis-acting elements of the 1500 bp upstream sequence of *BnaMYB* promoters in order to determine the potential function of the *BnaMYB* genes, excluding components with uncertain functions and general transcriptional regulatory elements (Table 2, Figure 6). All details of cis elements of each gene are listed in Supplementary Table S5. ABRE, the AuxRR core, a TGA element, a TGA box, a TGACG motif, a CGTCA motif, a P box, a GARE motif, TCA and a TCA element were distinguished as being responsive elements related to four phytohormones (abscisic acid (ABA), auxin, methyl jasmonate (MeJA), gibberellin (GA), and salicylic acid (SA)). Overall, we focused on and identified eight types. We also found four stress-related (drought, low-temperature, anaerobic, defense, and stress) responsive elements, including MBS, MYB, MYC, LTR, ARE and TC-rich repeats, suggesting their involvement in stress responses. The drought stress-responsive elements MBS, MYC and MYB were primarily found in 13 genes, 31 genes and 35 genes, respectively. These findings revealed that the majority of *BnaMYB* genes have drought-responsive elements, indicating that *BnaMYB* genes may be extremely important in responding to the stress caused by drought.

### 2.8. Tissue-Specific Expression Patterns of BnaMYB Genes

**Figure 7:**
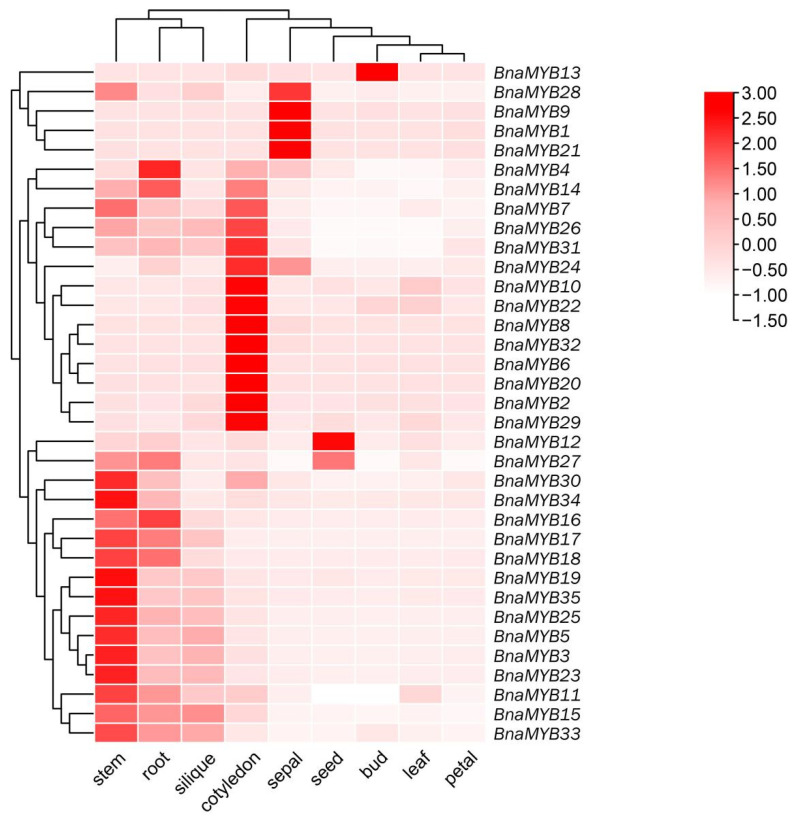
Heat map showing the expression profiles of *BnaMYB* genes in different tissues. The sampling time for the seeds, leaves, and siliques was the 10th day. The red, pink, and white colors display high to low expression levels. The heat map of expression was created by taking the log10 of transcripts per million (TPM). The transcriptomic data were sourced online from the BnTIR: *Brassica napus* information resource (http://yanglab.hzau.edu.cn, accessed on 2 May 2022).

Through the use of RNA-seq data from *Brassica napus* (ZS11 variant) (BioProject ID: PRJCA001495), the histo-specific expression profiles of *BnMYB* genes were detected in nine different tissues and organs (i.e., stems, roots, siliques, cotyledons, sepals, seeds, buds, leaves, and petals). The expression patterns of all *BnaMYB* genes varied in the different tissues, as shown in Figure 7 and Table S6. For instance, the majority of genes displayed greater expression levels in the stems, including *BnaMYB3*, *BnaMYB5*, *BnaMYB7*, *BnaMYB11*, *BnaMYB14*, *BnaMYB15*, *BnaMYB16*, *BnaMYB17*, *BnaMYB18*, *BnaMYB19*, *BnaMYB23*, *BnaMYB25*, *BnaMYB26*, *BnaMYB27*, *BnaMYB28*, *BnaMYB30*, *BnaMYB31*, *BnaMYB33*, *BnaMYB34* and *BnaMYB35.* In the cotyledons, a few genes showed higher expression levels, including *BnaMYB2*, *BnaMYB4*, *BnaMYB6*, *BnaMYB7*, *BnaMYB8*, *BnaMYB10*, *BnaMYB14*, *BnaMYB20*, *BnaMYB22*, *BnaMYB24*, *BnaMYB26*, *BnaMYB29*, *BnaMYB31* and *BnaMYB32.* In the roots, *BnaMYB4*, *BnaMYB11*, *BnaMYB14*, *BnaMYB15*, *BnaMYB16*, *BnaMYB17*, *BnaMYB18*, *BnaMYB27*, *BnaMYB31* and *BnaMYB33* displayed higher expression patterns. In the sepals, *BnaMYB1*, *BnaMYB9*, *BnaMYB21* and *BnaMYB28* exhibited higher expression patterns. In the siliques, *BnaMYB5*, *BnaMYB15* and *BnaMYB33* displayed higher expression patterns. Likewise, in the seeds, many genes, including *BnaMYB12* and *BnaMYB27*, showed higher expression patterns. These findings led us to the conclusion that the *BnaMYB* gene family was essential for rapeseed’s development at all stages, and that other members with similar expression traits might serve related purposes.

### 2.9. Expression Profiles of BnMYB Genes under Drought Stress

**Figure 8:**
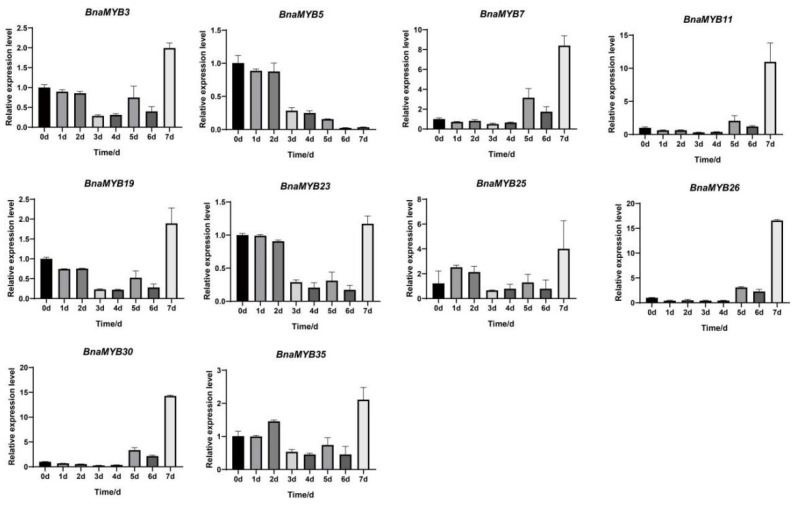
The expression patterns of *BnaMYB* genes under drought conditions. The 0 h (CK), 2, 4, 6, and 8 d labels indicate the time points (days) when the samples were harvested for studying gene expression after the drought treatment.

The majority of the *BnaMYB* genes had drought-responsive elements, which suggests that they may be involved in the response to drought, according to an analysis of the cis-acting elements in the upstream sequence of the *BnaMYB* promoters we conducted before. We chose 10 lignin-related genes (*BnaMYB3*, *BnaMYB5*, *BnaMYB7*, *BnaMYB11*, *BnaMYB19*, *BnaMYB23*, *BnaMYB25*, *BnaMYB26*, *BnaMYB30* and *BnaMYB35*) and submitted the 10 genes to drought treatment for 7 days to further discover their potential function (Figure 8). The primers used for gene expression analysis are shown in Table S7.

The expression patterns of *BnaMYB* genes under drought stress showed three patterns. First, the expression of *BnaMYB3*, *BnaMYB19*, *BnaMYB23*, *BnaMYB25* and *BnaMYB35* decreased gradually under the drought treatment and then decreased to the lowest level at 3 d or 4 d, then reached the peak on Day 7, showing a U-shaped curve. However, the expression of *BnaMYB5* decreased gradually after 0 h and reached the lowest point at 6 d and 7 d. Lastly, the expression levels of *BnaMYB7*, *BnaMYB11*, *BnaMYB26*, and *BnaMYB30* were very low in the first 4 days, significantly increased on Days 5 and 6, and rapidly increased on Day 7 to reach the peak, indicating that drought treatment could activate the expression of these four genes.

The majority of *BnaMYB* genes generally had various drought expression patterns, indicating that these *MYB* genes were engaged in the pathway that causes drought stress. Four genes (*BnaMYB7*, *BnaMYB11*, *BnaMYB26* and *BnaMYB30*) showed significantly upregulated expression under drought stress.

## 3. Discussion

The *R2R3-MYB* gene family of *B. napus* and related species was biologically analyzed in this work, including their geographic position, phylogenetic analysis, gene structure, homologous protein domains, and predicted 3D protein structure. The findings demonstrated that the 19 rapeseed chromosomes contained an unequal distribution of 35 *BnaMYB* family members. These individuals were divided into three groups through phylogenetic analysis, and the majority of the genes within each subgroup shared similar gene structures, conserved protein domains, and three-dimensional protein structures, which is indicative of the *BnaMYB* gene family’s evolutionary stability.

### 3.1. Characterization and Evolution of the R2R3-MYB Gene Family in Rapeseed

Allotetraploid rapeseed is a crop that has been engaged in extensive genome replication and combination activities [35]. One of the biggest obstacles to agricultural production in the modern world is dry soils. Crop plants have been bred conventionally and genetically to increase tolerance or resistance to drought and salinity in an effort to boost agricultural productivity in impacted areas. Future breeding and genetic engineering plans are likely to be greatly aided by a knowledge of the genomic mechanisms underlying plants’ responses to drought stress [36].

In plants, the *MYB* family has a significant function. The function of *MYBs* has been systematically researched in *Arabidopsis* [16,37], *Setaria italica* (Muthamilarasan et al., 2014), *Vitis vinifera* [38], *Zea mays* [25], *Populus trichocarpa* [39], *Gossypium raimondii* [40], and other plants, especially *Arabidopsis* and *Oryza sativa* [24]. The largest *MYB* gene subfamily in plants is the *R2R3-MYB* subfamily. Genetic methods have been used to characterize a large number of R2R3-MYB proteins, and it has been discovered that these proteins are involved in the regulation of a variety of plant-specific processes, such as (i) primary and secondary metabolism, (ii) cell fate and identity, (iii) developmental processes, and (iv) responses to biotic and abiotic stresses [16]. To the best of our knowledge, the rapeseed genome does not yet have a complete description of the *R2R3-MYB* gene family. The availability of rapeseed’s genome sequences makes it possible to identify rapeseed genes across the entire genome [35]. Here, we identified 35 *BnaMYB* genes in the genome of rapeseed.

The *BnaMYB* gene family may have developed in the rapeseed genome through segmental events and many tandem duplications. A key strategy for gene expansion is tandem duplication. Genes that have been enlarged by tandem duplication are always dispersed throughout chromosomes as a group [41,42]. Exon-intron connections and numbers, in addition to duplication events, can help to explain how the gene family has evolved [43,44,45]. When the gene structures of *BnaMYBs* and members of the same group were studied, it was shown that *MYB* genes contributed equally to exon/intron allocation in terms of exon and intron numbers, while *BnaMYBs* showed similar motif patterns. These findings suggest that these *R2R3-MYB* members could play similar roles in a range of abiotic stressors.

### 3.2. Diversity in the Functional Expression of BnaMYB Genes in Brassica napus

We looked at the expression patterns of 35 *BnaMYB* genes in diverse organs using publicly accessible data. The results showed that they varied in their patterns of expression in different tissues and that they were expressed in different tissues. In stems and cotyledons, 85.7% of *BnaMYB* genes were primarily expressed, while the primary expression of 10 genes was detected in the roots. *BnaMYB12* was only strongly expressed in the seeds and less so in other tissues. *BnaMYB1*, *BnaMYB9*, *BnaMYB21* and *BnaMYB28* displayed higher transcription levels in the sepals. These findings suggest that the *BnaMYB* gene family might well be important for rapeseed’s development and expansion at all stages. Twenty of the *BnaMYB* genes were mostly expressed in the stems; therefore, they may play a crucial role in how the stems react to environmental influences. Little research has been conducted on *Brassica napus*, despite the fact that several *R2R3-MYB* genes have been linked to drought adaptations and endurance in some species, particularly in rice and *Arabidopsis* [46,47,48,49]. Hence, our study of *BnaMYBs* is helpful to analyze the response of *R2R3-MYB* to drought stress in *Brassica napus*.

Numerous members of the plant *R2R3-MYB* family have been identified as transcription factors that are triggered by abiotic stress to date [47,50,51]. Cotton’s resilience to drought can be improved by transgenic *Arabidopsis* overexpressing the *R2R3-MYB* gene *GaMYB85* [52]. *Arabidopsis* overexpressing *LpMYB1* is more tolerant to salt and drought because of this R2R3-MYB factor [53]. Under prolonged drought stress, *TaMYBsdu1* increases noticeably in the roots and leaves of wheat [47]. *TaMYB33* improves tolerance to salt and drought, partly by restoring the osmotic equilibrium and eliminating superoxide radicals [54,55]. *TaMYB30-B* and *TaMYB19-B* can increase the ability of transgenic *Arabidopsis* plants to withstand drought stress [56,57]. These studies indicate that *R2R3-MYB* has the function of dealing with drought stress in wheat, cotton, and *Arabidopsis* and their function during drought stress is affected by the ABA synthesis and signal transduction pathway. For instance, the accumulation of 35S:*GaMYB85* resulted in the transcription of *RD22, RD29A, ADH1, AB15,* and *P5CS*. The *AB15* gene, which codes for a bZIP TF, is known to be an ABA-responsive gene that is important for seed germination under drought stress [58,59]. In light of the previous analysis, we speculated that drought stress in *Brassica napus* is affected by the lignin content. MYB46 controls not only the signaling molecules but also the genes that produce all three of the primary components of the secondary walls: grain, hemicellulose, and lignin [60]. Additionally, *MYB85* controls the production of lignin by regulating the lignin-synthesizing genes and, when overexpressed, results in ectopic lignin deposition [61]. This indicates that the *BnaMYB* gene family may affect lignin synthesis, and lignin synthesis responds to drought stress. We will pay attention to this in future research to deeply understand the relationship between the *BnaMYB* gene family and lignin.

The tissue-specific expression patterns reflected which tissues of the plant the gene mainly acts on. We found that 20 genes were highly expressed mainly in the stem, indicating that these genes were probably involved in lignin synthesis. Ten of these genes were selected for drought treatment, and their expression profiles were detected under drought stress. Our RT-qPCR data showed that the *BnaMYB* gene had significant changes under drought conditions, which proved that 10 genes were responsive to drought stress. The expression of *BnaMYB5* decreased significantly under drought stress, indicating that this gene could not cope with drought; *BnaMYB3*, *BnaMYB19*, *BnaMYB23*, *BnaMYB25* and *BnaMYB35* showed U-shaped curves, indicating that these five genes did not show drought resistance under mild drought stress but showed drought resistance under severe drought. Four genes (*BnaMYB7*, *BnaMYB11*, *BnaMYB26* and *BnaMYB30*) were significantly upregulated under drought stress, indicating that they are probably drought-resistant genes. According to our understanding, this is the first evidence indicating that *R2R3-MYB* genes in *B. napus* are implicated in the response to drought stress. This work is helpful for understanding the characteristics and functions of *R2R3-MYB* genes in different species.

## 4. Methods

### 4.1. Identification of Rapeseed’s R2R3-MYB Family Members

On the BnPIR website (http://cbi.hzau.edu.cn/bnapus/, accessed on 14 February 2022) [35], the genome sequences, protein sequences, and gene annotation data of rapeseed were downloaded. The Markov model of the conserved domains of *R2R3-MYB* (Myb_DNA-binding, PF00249) was downloaded from the Pfam database (http://pfam.xfam.org/, accessed on 14 February 2022). The cut-off E-value was chosen to 1e-10 when using the Markov model on HMMER software to preliminary screen the protein sequences of rapeseed. In order to exclude possible R2R3-MYB proteins without two conserved MYB DNA-binding domains, all candidate proteins were then uploaded to three online sources, SMART (http://www.omicsclass.com/article/681, accessed on 17 February 2022), NCBICDD (http://www.omicsclass.com/article/310, accessed on 17 February 2022), and PFAM (http://pfam.xfam.org/, accessed on 17 February 2022). For the MW and pI prediction analyses, ExPASy (http://web.expasy.org/protparam, accessed on 18 February 2022) received the identified *R2R3-MYB* candidate genes. Additionally, the subcellular localization was predicted by WoLF PSORT (https://www.genscript.com/wolf-psort.html, accessed on 18 February 2022).

### 4.2. Chromosome Location and Phylogenetic Analysis

The BnPIR website (http://cbi.hzau.edu.cn/bnapus, accessed on 5 March 2022) provided information on the chromosome location of *BnaMYBs*. After that, the distribution status of the *BnaMYBs* discovered on the chromosomes was shown using MapChart software. With the aid of Adobe Illustrator, the outcomes were improved.

We downloaded the protein sequences of the MYB family members of *Brassica oleracea*, *Brassica rapa*, *Capsella rubella*, *Oryza sativa*, *Raphanus sativus* and *Zea mays*. The sequences of the protein on the *Brassica oleracea* and *Brassica rapa* were collected from the Phytozome website (https://phytozome.jgi.doe.gov/pz/portal.html, accessed on 10 March 2022). The sequences of the protein in *Oryza sativa* and *Zea mays* were collected from the Ensembl website (http://plants.ensembl.org/index.html, accessed on 10 March 2022). The protein sequences of *Capsella rubella* and *Raphanus sativus* were collected from the NCBI website (https://www.ncbi.nlm.nih.gov/, accessed on 10 March 2022). OrthoMCL software (v2.0.3) [62] was used to search for orthologous, co-orthologous, and paralogous genes in *Arabidopsis thaliana*, *Brassica oleracea*, *Brassica rapa*, *Capsella rubella*, *Oryza sativa*, *Raphanus sativus* and *Zea mays* using entire R2R3-MYB protein sequences. The E-value cut-off of an all-against-all BLASTP alignment process was set at 1 × 10^−10^, and the alignments with a match cut-off value lower than 50 were eliminated.

These plants’ MYB protein sequences were analyzed using Clustal W after the protein sequences of *MYB* family members from *B. napus*, *Arabidopsis*, *B. oleracea*, *B. rapa*, *Capsella rubella*, *Oryza sativa*, *Raphanus sativus* and *Zea mays* were retrieved. Additionally, MEGA 11.0 software was used to create the evolutionary tree using the neighbor joining method (NJ) once the results of sequence alignment were obtained [63,64]. Finally, we used Evolview (http://www.omicsclass.com/article/671, accessed on 13 March 2022) to visualize the evolutionary tree.

### 4.3. Distinct Gene Structure and Conserved Protein Domain of R2R3-MYBs

The gene structure of the *R2R3-MYB* genes contains intron and exon information [65]. MEME Suite (https://meme-suite.org/meme/, accessed on 30 March 2022) was used to examine the homologous domains of the genes using the BnaMYB protein sequences, increasing the maximum motif count to 10, the maximum motif amino acid count to 20, and the minimum motif width to 6, and leaving all other variables at their normal values. Finally, the similar motifs of BnaMYB proteins were viewed using TBtools software.

### 4.4. Predicted 3D Structure of R2R3-MYB Proteins

Thirty-five BnaMYB protein sequences were entered into the DNAMAN8.0 program for triple sequence comparison. We next predicted the 3D structure of the proteins using the internet tool Phyre2 (http://www.sbg.bio.ic.ac.uk/phyre2/html/page.cgi?id=index, accessed on 25 April 2022).

### 4.5. Analysis of Collinearity within B. napus and Related Species

McScanx analysis was used to analyze the *BnaMYB* genes’ intra-species collinearity in *B. napus* and Circos software (v0.69-8) was used to visualize the associations. Additionally, the McScanX software (Python version) was used to visualize the collinearity.

### 4.6. Analysis of the Synteny of BnMYB Genes

The CDS sequences of 35 *BnaMYB* genes were input into DNAMAN8.0 for sequence alignment. We selected the CDS sequences of homologous gene pairs, uploaded them to the ALTER website (http://www.sing-group.org/ALTER/, accessed on 28 January 2023), and outputted the results in ALN format. We converted ALN format to AXT format with AXT Converter and used KaKs Calculator 2.0 software to calculate the value of Ka/KS.

### 4.7. Cis-Acting Elements and Functional Annotation

The publicly available online whole-genome information on *B. napus* (http://cbi.hzau.edu.cn/bnapus, accessed on 28 April 2022) provided 1500 bp of the upstream sequences of the *BnaMYB* genes. The cis-acting components were extracted using the online tool Plant CARE (http://bioinformatics.psb.ugent.be/webtools/plantcare/html, accessed on 29 April 2022) and then visualized using the online tool DSGS.

### 4.8. Spatial and Temporal Expression Patterns of R2R3-MYB Genes

The BnTIR: *Brassica napus* genomic resource is a website (http://yanglab.hzau.edu.cn, accessed on 2 May 2022) [66]. The stems, roots, siliques, cotyledons, sepals, seeds, buds, leaves, and petals were among the tissues for which we obtained RNA-seq data. To create the heat map of expression, the data were entered into the Heatmapper program (http://www.heatmapper.ca, accessed on 2 May 2022).

### 4.9. Plant Materials and Treatment Methods

In this study, the rapeseed genotype “ZS11”, a typical cultivated variety, was used for the stress treatments. The seeds of the ZS11 genotype were furnished by OCRI, CAAS, China. Before the stress treatments, some seeds were randomly selected from the same batch of seeds to determine the germination rate. The seeds with a 100% germination rate were considered to be vigorous seeds. The vigorous seeds were carefully chosen and sterilized with a 10% hypochlorous acid solution for 5 min. The seeds were grown on water-saturated filter paper in a chamber (25 °C day and night, and a 16 h/8 h light/dark cycle) until the plants grew to the five-leaf stage. When the plants had grown to the five-leaf stage, we stopped watering to impose drought stress. Samples from the stem were collected 0, 1, 2, 3, 4, 5, 6 and 7 days after the initiation of the drought treatment [67,68]. Moreover, three biological replicates of each sample were collected. The gathered stems were immediately put into liquid nitrogen and kept in a freezer for later use at −80 °C.

### 4.10. RNA Extraction and RT-PCR Analysis

Using a carbohydrate phenolic total RNA extraction kit, the total RNA was isolated from leaves that had undergone various drought treatments (Tiangen Biochemical Technology Co., Ltd.). An ultramicroscopic spectrophotometer was used to measure the quantity and quality of the RNA (Thermo Fisher, Waltham, MA, USA, Nanodrop One). As samples for subsequent RT-qPCR experiments, we synthesized cDNA using a reverse transcription kit and diluted it 100 times with ddH_2_O. Specific primers were created using the online qPCR Primer Database (https://biodb.swu.edu.cn/qprimerdb/, accessed on 27 May 2022) based on the coding sequences of the *BnaMYB* genes. The specific primers were amplified, and the primers with a ct value of no more than 30, a normal amplification curve, and no obvious impurity peak in the dissolution curve were selected for subsequent experiments. The quantitative experiment was conducted in real-time using SYBR Premix Ex TaqTM (TaKaRa). Three separate replicates were collected for this experiment, and the samples that had not been subjected to drought were used as standards. The 2^−ΔΔCT^ technique is often used in investigations of the relative expression of genes [69]. The graphs were developed using GraphPad Prism 9.0.0 software.

## 5. Conclusions

In total, 35 *BnaMYB* genes were found in *B. napus*, dispersed unevenly across 19 chromosomes. We analyzed how the MYB proteins are related, their gene structure, their conserved motifs, and their three-dimensional structures. These genes were divided into three subfamilies, each of which had reasonably traditional gene architectures and patterns. The *BnaMYBs* promoter regions also contained cis-acting hormones and abiotic stress response components, along with drought-responsive genes. There were 48 pairs of large-segment repetitive genes discovered in *B. napus* through the analysis of collinearity. The *R2R3-MYB* genes of *B. napus* had undergone polyploidization and various degrees of loss and expansion, according to comparative genomic research. We also looked at the gene expression patterns of *BnaMYB* in various rapeseed tissues, and the results showed that the members of the *BnaMYB* gene family were crucial at different phases of the development of *B. napus*. In addition, we examined the expression patterns of 10 *BnaMYB* genes under drought. Overall, by carefully examining the conservation and divergence of the *BnaMYB* gene family’s activities, our work lays the biological groundwork for the subsequent functional discovery of *R2R3-MYB* genes in cruciferous plants.

## Figures and Tables

**Table 1:** The position and molecular information of *MYB* gene family in *B. napus*.

Gene Name	Gene ID	Chromosomes Position	CDS (bp)	Protein		
				Length (bp)	MW (kDa)	pI
*BnaMYB1*	BnaA01G0357100ZS	32256891-32258168(+)	1074	358	108816.87	5.01
*BnaMYB2*	BnaA01G0365200ZS	32786604-32787938(+)	885	295	112433.86	5.06
*BnaMYB3*	BnaA02G0045300ZS	2546354-2548355(-)	840	280	167892.18	4.92
*BnaMYB4*	BnaA02G0378200ZS	33155658-33156863(-)	1011	337	102768.22	5.06
*BnaMYB5*	BnaA03G0050300ZS	2408564-2410538(-)	843	281	166460.81	4.9
*BnaMYB6*	BnaA03G0135900ZS	6915762-6916844 (-)	804	268	91357.94	5
*BnaMYB7*	BnaA03G0312000ZS	16483091-16484321 (-)	1023	341	104361.56	4.96
*BnaMYB8*	BnaA03G0487400ZS	27023236-27024166 (+)	753	251	77433.37	5.11
*BnaMYB9*	BnaA05G0412600ZS	39809095-39810464 (+)	1047	349	116330.89	5.01
*BnaMYB10*	BnaA05G0423500ZS	40421096-40422152 (+)	885	295	88892.42	5.08
*BnaMYB11*	BnaA05G0452900ZS	41865529-41866766 (+)	1008	336	102341.73	5.07
*BnaMYB12*	BnaA06G0057600ZS	3572905-3574580 (+)	996	332	142233.5	4.97
*BnaMYB13*	BnaA06G0163800ZS	10091574-10092862 (-)	777	259	110035.56	4.97
*BnaMYB14*	BnaA06G0344100ZS	42258187-42259525 (-)	1071	357	112803.96	5.05
*BnaMYB15*	BnaA09G0021000ZS	1330928-1332132 (-)	1011	337	100979.61	4.97
*BnaMYB16*	BnaA09G0060200ZS	3683531-3684746 (+)	1008	336	101112.82	5.06
*BnaMYB17*	BnaA09G0135900ZS	8220775-8222850 (-)	1110	370	175135.25	4.93
*BnaMYB18*	BnaA09G0147200ZS	8944567-8946278 (+)	1110	370	143838.31	5
*BnaMYB19*	BnaA10G0226500ZS	23076072-23078201 (+)	843	281	179215	4.96
*BnaMYB20*	BnaC01G0191600ZS	14174638-14175619 (+)	807	269	82001.37	5.1
*BnaMYB21*	BnaC01G0443900ZS	50997955-50999227 (+)	1074	358	108353.21	5.02
*BnaMYB22*	BnaC01G0455800ZS	52367618-52368963 (+)	885	295	113600.18	5.05
*BnaMYB23*	BnaC02G0052100ZS	3261244-3263562 (-)	837	279	195053.44	4.89
*BnaMYB24*	BnaC02G0506000ZS	61058311-61059530 (-)	1023	341	103681.14	5.06
*BnaMYB25*	BnaC03G0058300ZS	3046411-3048356 (-)	840	280	163856.16	4.91
*BnaMYB26*	BnaC03G0373800ZS	25163139-25164386 (-)	1023	341	105884.4	4.96
*BnaMYB27*	BnaC05G0072600ZS	4097294-4098491 (+)	1005	335	100976.31	5.02
*BnaMYB28*	BnaC05G0464600ZS	51456439-51457659 (+)	987	329	103719.42	5.04
*BnaMYB29*	BnaC05G0475500ZS	52295048-52296128 (+)	885	295	90920.47	5.08
*BnaMYB30*	BnaC05G0511100ZS	54454866-54456100 (+)	1008	336	102098.47	5.07
*BnaMYB31*	BnaC07G0351100ZS	48234276-48235576 (+)	1074	358	108612.43	5.04
*BnaMYB32*	BnaC07G0465900ZS	56085074-56086043 (+)	786	262	80884.41	5.09
*BnaMYB33*	BnaC09G0004300ZS	266734-267940 (-)	1011	337	101103.74	4.97
*BnaMYB34*	BnaC09G0048600ZS	3138803-3140038 (+)	1017	339	103080.97	5.06
*BnaMYB35*	BnaC09G0531900ZS	62845844-62847979 (+)	837	239	179779.67	4.96

**Table 2:** The *cis*-elements identified in more than three *BnaMYB* genes.

Site Name	Sequence	Function of the Cis-Elements
TGA element	AACGAC	Auxin-responsive element
TC-rich repeats	GTTTTCTTAC	Cis-acting element involved in defense and stress responses
LTR	CCGAAA	Cis-acting element involved in low-temperature responses
TCA element	CCATCTTTTT	Cis-acting element involved in salicylic acid responses
ABRE	ACGTG	Cis-acting element involved in abscisic acid responses
AuxRR core	GGTCCAT	Cis-acting regulatory element involved in auxin responses
CGTCA-motif	CGTCA	Cis-acting regulatory element involved in the MeJA responses
TGACG motif	TGACG	Cis-acting regulatory element involved in MeJA responses
GARE motif	TCTGTTG	Gibberellin-responsive element
P box	CCTTTTG	Gibberellin-responsive element
MBS	CAACTG	MYB binding site involved in drought response
TGA box	TGACGTAA	Part of an auxin-responsive element
ARE	AAACCA	Cis-acting regulatory element essential for anaerobic induction
MYB	TAACCA	Drought response element
MYC	CAATTG	Drought and MYBd reaction element
TCA	TCATCTTCAT	Cis-acting element involved in salicylic acid responses

## Data Availability

All data generated or analyzed during this study are included in this published article and its Supplementary Materials. Zhongshuang11 is an elite conventional variety of rapeseed bred and preserved by OICR-CAAS (Oil Crops Research Institute).

## Supplementary Materials

The following supporting information can be downloaded at: https://www.mdpi.com/article/10.3390/plants12040886/s1, Figure S1: Multiple sequence alignment of BnaMYB proteins; Figure S2: Three-dimensional structure prediction of Myb_DNA-binding domain of BnaMYB proteins. The images are from dark blue to dark red, indicating from the N end to the C end; Figure S3: CDS sequence alignment of 35 BnaMYB genes; Table S1: The position of genes in chromosomes; Table S2: Protein sequence of MYB genes in each species; Table S3: The information of identified 10 motifs in BnMYB proteins; Table S4: Information on Ka, Ks and Ka/Ks ratios of Brassica napus. See the abbreviations at the end of the table [70]; Table S5:Cis-element of genes; Table S6: Absolute gene expression values in thirty-five BnaMYBs; Table S7:Primers used for gene expression analysis.
