# Supplementary figures and images for: Identification and Phylogenetic Analysis of the R2R3-MYB Subfamily in Brassica napus
Source: Plants (Basel). 2023 Feb 16;12(4):886. doi: 10.3390/plants12040886 (PMC9962269; doi:10.3390/plants12040886)

## Slide 1
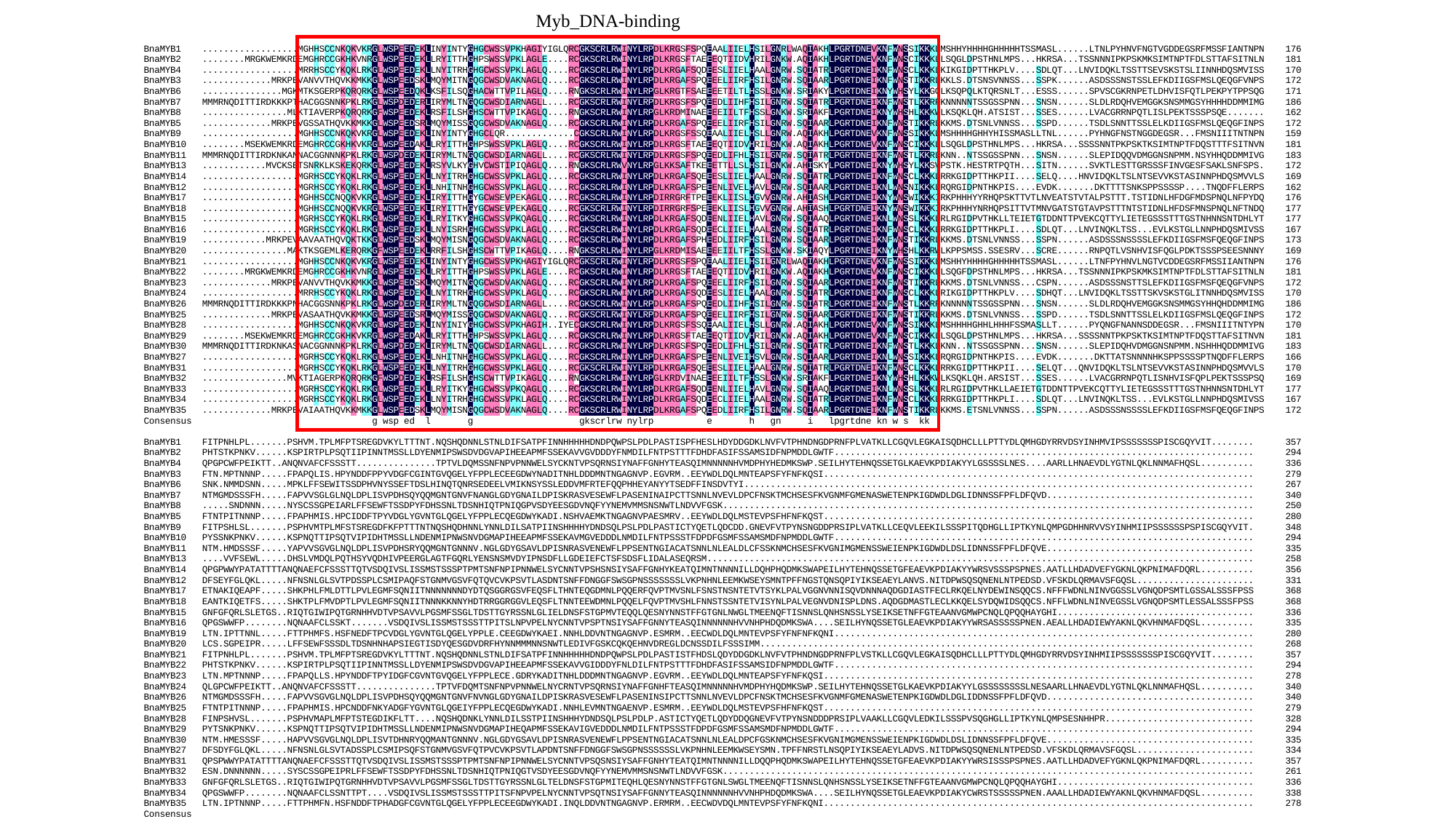

Myb_DNA-binding

Supplement: Supplementary file 1 [file plants-12-00886-s001.zip › plants-2182704-supplementary/Figure S1.pptx]

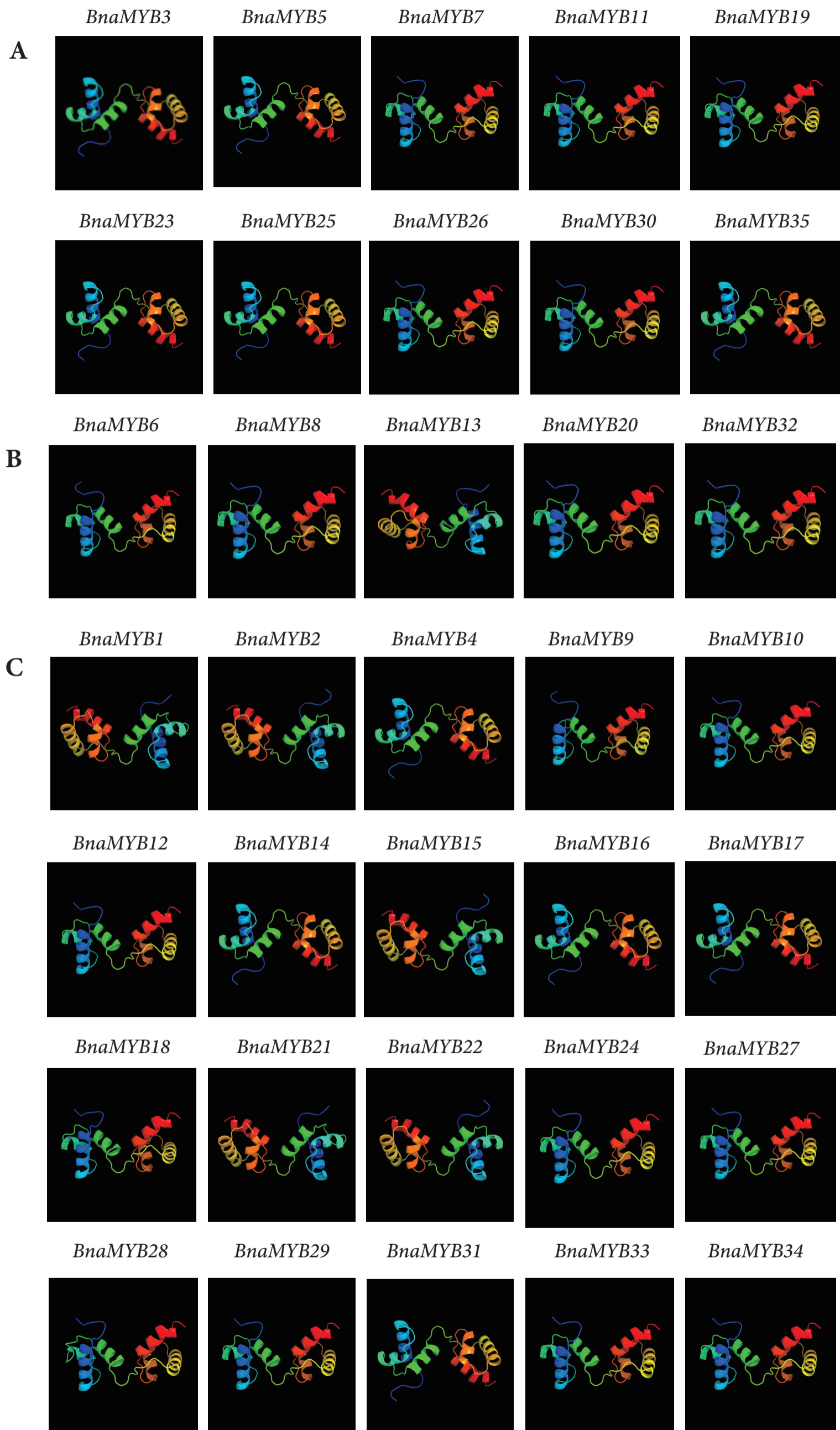

Supplement: Supplementary file 1 [file plants-12-00886-s001.zip › plants-2182704-supplementary/Figure S2.pdf]
